# Tuberculose pseudo tumorale du col de l'utérus

**DOI:** 10.11604/pamj.2019.32.163.17763

**Published:** 2019-04-09

**Authors:** Michel Assane Ndour, Maimouna Sow, Ibou Thiam, Boundia Djiba, Fabrice Senghor, Nafi Diagne, Awa Cheikh Ndao, Abdoulaye Pouye

**Affiliations:** 1 Service de Médecine Interne, Centre Hospitalier Aristide le Dantec, Dakar, Sénégal; 2 Service d'Anatomopathologie, Centre Hospitalier Aristide le Dantec, Dakar, Sénégal

**Keywords:** Tuberculose, col cervical, Dakar, Tuberculosis, cervix, Dakar

## Abstract

La localisation cervicale de la tuberculose est rare et peut prendre l'apparence d'un cancer du col utérin. La présentation pauci-symptomatique et l'évolution insidieuse sont à l'origine d'un retard diagnostique. Les symptômes communément rencontrés sont non spécifiques, ce qui contribue au retard thérapeutique et majore le risque d'infertilité qui reste la séquelle quasi inéluctable. Nous rapportons un cas de tuberculose du col utérin dont le diagnostic de prime abord n'a pas été évident. En effet une patiente a été adressée à notre département pour suspicion de cancer du col utérin. Le diagnostic de suspicion de cancer a été alors retenu devant un col qui saignait au contact avec une tomodensitométrie abdominopelvienne en faveur d'une tumeur du col utérin. Une biopsie de confirmation histologique a été indiquée. On a retrouvé à l'examen anatomopathologique, un granulome épithélio-giganto-cellulaire avec nécrose caséeuse, compatible avec une tuberculose cervicale. La recherche d'un terrain était négative. Un traitement antituberculeux instauré a conduit à la guérison. Retenue souvent sur la base d'éléments présomptifs, la décision diagnostique et thérapeutique de tuberculose du col cervicale reconnait la place de l'examen anatomopathologique.

## Abstract (en)

Tuberculosis of the cervix is rare and can mimick cervical cancer. Patients are paucisymptomatic and the disease is characterized by an insidious evolution, hence the delay in diagnosis. Common symptoms are non-specific contributing to therapeutic delay and increasing the risk of infertility which is perhaps inevitable. We report a case of tuberculosis of the cervix whose diagnosis given first wasn't obvious. Indeed, a patient was referred to our Department with suspected cervical cancer. Suspected diagnosis of cancer was then retained based on the presence of vaginal bleeding on contact and abdominopelvic CT scan results showing cervical cancer. Biopsy was indicated in order to confirm the diagnosis histologically. Anatomo-pathological examination objectified epitheliogigantocellular granuloma with caseous necrosis, supporting cervical tuberculosis. The other examinations were negative. The patient received TB treatment which led to healing. Diagnosis and treatment of tuberculosis of the cervix is often based on presumptive elements, hence the importance of anatomo-pathological examination.

## Introduction

La tuberculose est une maladie consécutive à une infection par des bacilles du complexe *Mycobacterium tuberculosis* qui comprend Mycobacterium tuberculosis stricto sensu, *M. bovis*, *M. africanum* et d'autres espèces mycobactériennes plus rarement rencontrées en pathologie humaine. Les localisations sont multiples. La suspicion clinique de la tuberculose du col utérin ne se fait pas généralement en première intention, du fait de sa rareté et de sa similitude avec le cancer du col, maladie fréquente et souvent dépistée dans notre contexte [1]. Du fait de la localisation cervicale, la découverte de cette pathologie est souvent différée par un diagnostic différentiel large. Dans le cas que nous rapportons, nous insistons sur l'importance de l'examen histologique dans la démarche diagnostique en attirant l'attention du praticien sur le fait que tout col rouge qui saigne n'est pas nécessairement un cancer du col

## Patient et observation

**Figure 1:**
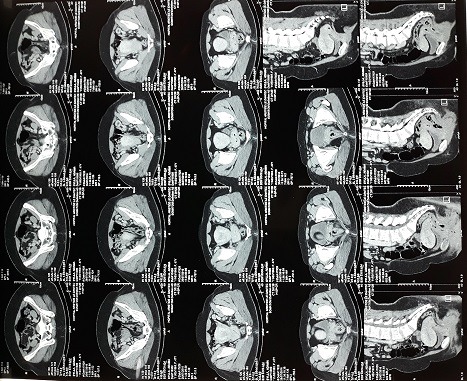
TDM abdominopelvienne avec injection, en reconstruction sagittale, passant par l'utérus montrant la lésion cervicale

**Figure 2:**
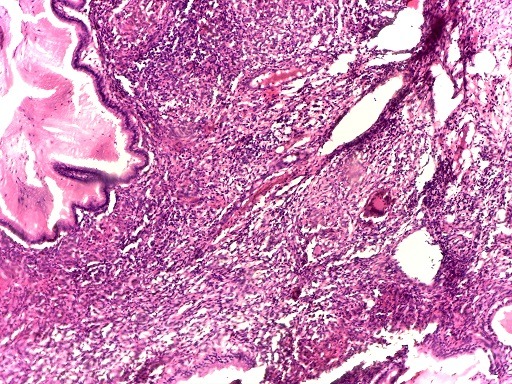
Muqueuse endocervicale siège d'un infiltrat inflammatoire granulomateux épithélio-giganto-cellulaire

**Figure 3:**
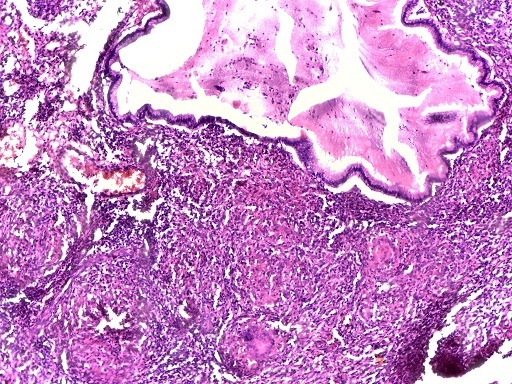
Muqueuse endocervicale siège d'un granulome tuberculeux (granulome épithélio-giganto-cellulaire associé à des foyers de nécrose caséeuse débutante)

Nous vous présentons une femme de 48 ans sans antécédents pathologiques particuliers qui a consulté il y'a 2 ans pour métrorragies. Une vaccination au BCG a été faite et nous avons noté une notion de contage tuberculeux dans la famille. A l'examen physique, nous avons objectivé des métrorragies qui survenaient de manière spontanées, associées à des douleurs pelviennes d'évolution intermittentes, des leucorrhées non fétides. Au toucher vaginal, le col était dur, légèrement sensible et le doigtier souillé de sang. L'examen au spéculum montrait un col augmenté de volume, épaissi et induré sans ulcération, à bords irréguliers, saignant au contact. Le reste de l'examen est sans particularités. Deux échographies pelviennes ont été réalisées mais ne décelant aucune anomalie. Une tomodensitométrie abdominopelvienne a été faite retrouvant un col utérin modérément épaissi avec des contours antérolatéraux irréguliers, postéro latéraux irréguliers en rapport avec l'infiltration des paramètres, un épaississement modéré du 1/3 supérieur du vagin, une absence d'infiltration suspect des structures urinaires et digestive pelviennes, des adénopathies iliaques bilatérales et retro péritonéales, une absence de localisation néoplasique, séquelles bilatérales de pyélonéphrite et concluait à une tumeur du col de l'utérus de stade T3bN1M0 (Figure 1). Cependant une première biopsie du col avec étude histologique pour confirmation trouvait une inflammation chronique non spécifique. Une seconde biopsie trouvait un granulome avec zone de nécrose faisant conclure à une tuberculose du col utérin. Elle montrait une muqueuse endocervicale siège d'un infiltrat inflammatoire granulomateux épithélio-giganto-cellulaire (Figure 2) et la nécrose (Figure 3). Une poursuite des explorations trouvait une intradermoréaction à la tuberculine phlycténulaire à 21 mm, une CRP positive à 12 mg. L'étude cytobactériologique des urines était normale. Des examens complémentaires à la recherche d'un terrain particulier étaient alors demandés: la radiographie des poumons et la radiographie du rachis dorsolombaire étaient normales, l'utérus et ses annexes étaient normaux à l'échographie pelvienne, la sérologie rétrovirale de l'immunodéficience humaine était négative. Sur la base des résultats des éléments cliniques, des éléments présomptifs paraclinique et surtout de l'examen anatomopathologique, un traitement antituberculeux est entrepris pour 6 mois, selon le protocole national. Au bout de quatre mois de traitement, les métrorragies ont disparu. La symptomatologie avait complètement régressé pour disparaitre trois mois plus tard.

## Discussion

Le diagnostic évoqué chez cette femme de 48 ans a été un cancer du col avant de retenir la tuberculose du col. Les résultats anatomopathologiques de la pièce de biopsie ont été un granulome épithélio-giganto-cellulaire avec nécrose caséeuse ce qui a permis de redresser le diagnostic. Le succès du traitement antituberculeux a conforté cette hypothèse. La tuberculose génitale féminine représente 6 à 10% de l'ensemble des localisations tuberculeuses. Les organes les plus fréquemment affectés sont les trompes, l'endomètre et les ovaires [2]. La localisation au niveau du col utérin est plutôt rare et représente 5% des localisations génitales [3].

Le mode de contamination varie selon les auteurs. Certains ont insisté sur la contamination sexuelle à partir d'une origine urogénitale masculine. Pour d'autres, l'atteinte du col pourrait être indirecte, par voie lymphatique, la première lésion causale étant en général guérie. Dans ce cas, sur le plan physiopathologique et en considérant le contexte épidémiologique, on pourrait suggérer une atteinte pulmonaire primitive, suivie d'une atteinte génitale secondaire. L'aménorrhée suggérerait une atteinte endométriale suivie ensuite d'une atteinte du col [4]. La tuberculose génitale est peu parlante cliniquement ou peut mimer d'autres pathologies, classiquement une néoplasie pelvienne (cancer de l'ovaire ou de l'endomètre, plus rarement vulvaire ou vaginal) ou un abcès pelvien ou péritonéal à pyogènes. Les signes cliniques révélant une localisation cervicale de la tuberculose sont peu spécifiques, associant des métrorragies provoquées ou spontanées, à des leucorrhées [5]. L'expression clinique du col, dans notre étude, est celui d'un col tuméfié, bosselé par endroit, saignant au contact, avec des leucorrhées blanchâtres, spumeuses. C'est ce qui a expliqué la confusion avec le cancer du col, d'autant plus qu'il n'y avait ni fièvre, ni perte de poids.

Certains auteurs rencontrant la même difficulté sont plus explicites quand ils déclarent que cliniquement la tuberculose du col ressemble au carcinome du col. Ils ajoutent que les mêmes symptômes comme un saignement vaginal anormal, un col pathologique avec des lésions bourgeonnantes peuvent être retrouvés dans la tuberculose du col. Mais s'il ne faut pas confondre tuberculose et cancer du col dans un souci de prise en charge, il faut savoir que les deux exceptionnellement peuvent être associés et des cas ont été rapportés [6]. La place de l'examen anatomopathologique dans la décision diagnostique et thérapeutique, dans notre étude a été déterminante. En effet l'examen histologique de notre prélèvement a retrouvé un granulome épithélio-giganto-cellulaire avec nécrose caséeuse ce qui nous a permis de retenir le diagnostic de tuberculose cervicale, en y associant les autres arguments présomptifs. La mise en évidence de bacilles acido-alcoolo-résistants à la coloration de Ziehl-Nielsen à l'examen histologique aurait davantage confirmé ce diagnostic. Mais selon certains auteurs, l'aspect histologique de granulome avec nécrose caséeuse typique seul suffit pour poser le diagnostic de tuberculose cervicale en l'absence de tout autre argument, d'autant plus qu'il ressort que dans un tiers des cas de tuberculose cervicale la culture est négative autorisant la mise en route d'un traitement médical d'épreuve [7]. Le diagnostic de tuberculose cervicale sans autre pathologie associé dans notre cas a été conforté par le fait que la recherche d'autres causes de lésion granulomateuse du type sarcoïdose ou parasitose s'est révélée négative, avec l'absence de corps étranger réfringent à la lumière polarisée et la négativité de la coloration au PAS.

Dans notre observation, le traitement antituberculeux pour 6 mois avec une régression spectaculaire au bout de 4 mois a confirmé davantage le diagnostic. Le recours au traitement chirurgical reste possible, initialement pour la prise en charge de complications (fistules ou abcès) ou secondairement en cas de résistance ou de rechute sous traitement médical bien conduit [8].

## Conclusion

Cette observation nous permet de rappeler la place importante de l'histologie dans le diagnostic de la tuberculose du col utérin. Bien que cette pathologie reste rare, il faut souvent y penser devant des lésions cliniques suspectes de cancer du col, et cela d'autant plus que la patiente présentait des métrorragies.

## Conflit d'intérêts

Les auteurs ne declarent aucun conflit d'intérêts.
